# Diagnostic and therapeutic impact of SPECT/CT in patients with unspecific pain of the hand and wrist

**DOI:** 10.1186/2191-219X-2-53

**Published:** 2012-09-28

**Authors:** Florian S Schleich, Maja Schürch, Martin W Huellner, Urs Hug, Urs von Wartburg, Klaus Strobel, Patrick Veit-Haibach

**Affiliations:** 1 Department of Radiology and Nuclear Medicine, Lucerne Cantonal Hospital, Lucerne, CH-6000, Switzerland; 2 Department of Hand and Plastic Surgery, Lucerne Cantonal Hospital, Lucerne, CH-6000, Switzerland; 3 Department of Medical Imaging, University Hospital Zurich, Rämistasse 100, Zurich, 8091, Switzerland

**Keywords:** SPECT/CT, Therapeutic impact, Multimodality imaging, Hand and wrist pain

## Abstract

**Background:**

Unspecific pain of the hand/wrist is a diagnostic challenge. Radiographs and planar bone scan are useful diagnostic tools in patients with unspecific wrist pain. Both modalities are deficient, either by not presenting metabolic disorders or due to inadequate anatomical resolution. Single photon emission computed tomography/computed tomography (SPECT/CT) claims to fuse both features.

**Methods:**

Fifty-one patients with persisting wrist pain were referred for evaluation by SPECT/CT. All patients received X-ray and early-phase/late-phase SPECT/CT imaging. SPECT/CT results were compared with X-ray alone and X-ray combined with planar bone scan. The therapeutic impact was evaluated in consensus with the referring hand surgeon.

**Results:**

A total of 48 lesions were detected on plain radiographs, 117 on planar bone scan, and 142 on SPECT/CT. SPECT/CT detected significantly more lesions than the other imaging modalities. In 30 out of 51 patients (61%), a positive concordance between the clinical diagnosis and SPECT/CT findings was found. In 19 out of 51 patients (37%), SPECT/CT findings had significant impact on consecutive therapy.

**Conclusions:**

SPECT/CT showed higher lesion detection rates compared to standard X-rays and planar bone scan. Significant impact on patient management could be demonstrated. SPECT/CT might be added to the workup of such a specific patient population when standard imaging fails to detect the patient's main pathology.

## Background

Unspecific pain of the hand and wrist is a diagnostic challenge, clinically for the hand surgeon and for diagnostic imaging. The low specificity of clinical symptoms causes difficulties in localizing the pain by means of clinical examination and imaging. Imaging results often do not correlate with clinical symptoms or fail to detect any significant lesion which could possibly explain the clinical symptoms. The primary diagnostic steps are generally plain radiographs in several views. Plain radiographs give a simple and useful overview over osseous changes, e.g., cysts as well as osteolytic and arthritic osseous changes. Secondary diagnostic steps are computed tomography (CT) and magnetic resonance imaging (MRI). MRI is able to detect pathologies of the cartilages, tendons, and sheaths, as well as the carpal ligaments. The bone is visualized as well, but the morphological structure of the carpal bones is more easily evaluable with CT, especially in the absence of bone marrow edema. However, both imaging techniques are limited in showing metabolic disorders. Another (also secondary) diagnostic step is bone scintigraphy and single photon emission computed tomography (SPECT) which is known to be a very sensitive but relatively unspecific imaging tool visualizing bone metabolism [1,2], e.g., in cases of bone matrix alteration in trauma or infection. Planar (two-dimensional) scintigraphy has a disadvantage concerning lesion localization due to the complex anatomy of the wrist. Modern SPECT/CT systems allow for detection of metabolic pathologies and exact concomitant anatomical localization which has significant therapeutic impact [3]. Additionally, the available CT component with high-resolution capabilities can provide additional diagnostic clarity of the metabolically active pathology and may detect additional, non-active pathologies.

So far, there is only very limited experience available concerning SPECT/CT in the evaluation of peripheral joints, and there is no experience in such a specific patient population [4]. Thus, the aims of the study were (1) to evaluate the diagnostic impact of SPECT/CT vs. standard imaging procedure (plain radiography and planar bone scan) in patients with unspecific pain of the hand and wrist and (2) to define the therapeutic impact of SPECT/CT in this specific patient population.

## Methods

### Patients

A total of 51 consecutive patients (31 females, 20 males, mean age 38 years, range 17 to 73 years, mean clinical follow-up after SPECT/CT 11 months) with persisting wrist pain after trauma or with chronic wrist/hand pain without trauma were evaluated retrospectively. The diagnosis of nonspecific wrist pain was made by the treating hand surgeon, based on patient history, clinical examination (based on clinical guidelines), and available imaging prior to SPECT/CT (see below) [5]. All patients were referred by their hand surgeon to our department for evaluation by SPECT/CT. Fifteen patients had wrist pain after trauma, of which six patients had a contusion, seven patients had a fracture, one patient had a supination trauma of the wrist, and one patient had a distortion trauma. All patients received planar radiographs (before SPECT/CT) as well as early-phase and late-phase planar bone scan of the wrist/hands and SPECT/CT (all combined within one procedure, named as SPECT/CT for the rest of the manuscript). Twenty-two patients had an additional MRI prior to SPECT/CT. Five patients had a CT-only examination prior to SPECT/CT. The mean time interval between the trauma or the first onset of chronic clinical symptoms and SPECT/CT was 14 months (range 1 to 49 months). Five patients had an interval of >24 months because the treating physician ordered serial MRI examinations prior to SPECT/CT imaging. However, all patients had chronic, clinically relevant symptoms at the time point of imaging, and conservative therapy failed to alleviate the symptoms. All patients gave written informed consent prior to the examination. Based on the retrospective nature of this study, IRB was waived.

### Imaging procedures

Planar bone scintigraphy was performed as early-phase (directly after injection) and late-phase (after 3 h) images on a dedicated, combined SPECT/CT system. SPECT/CT was performed on a hybrid SPECT/CT with a built-in flat-panel CT (BrightView XCT, Philips Healthcare, Best, Netherlands) after injection of 670-MBq (average) ^99m^Tc-DPD (technetium-99m-3,3-diphosphono-1,2-propanedicarboxylic acid, Teceos, Behringwerke AG, Marburg, Germany). Early-phase planar images were acquired directly after injection for 5 min (matrix 256 × 256, full field of view (FOV) 52 × 58 cm, LEHR collimator/for SPECT: 128 views over 360°, step-and-shoot noncircular acquisition). Late-phase SPECT/CT images were acquired after 3 h (matrix 512 × 512, 128 views over 360°, step-and-shoot noncircular acquisition, 20-s acquisition/step). CT images of the SPECT/CT were acquired in HI-REZ mode (120 kVp, 80 mA, slice thickness 0.33 mm, FOV 40 cm) and were reconstructed in all three planes with 1-mm slice thickness. The used parameters account for an additional CT dose of approximately 2.4 mSv. The used CT parameters represent a middle-dose CT rather than a high-dose standard CT. However, since the region of interest is in the periphery and the wrist does not have a high bone mass, such a middle dose is sufficient concerning image quality. Additionally, the achievable slice thickness and resolution based on the flat-panel technology are higher compared to a full diagnostic, standard high-dose CT with a typical maximal resolution of 0.6 mm. In all patients, FOV covered both wrists completely during planar and SPECT/CT imaging. Plain radiographs in three standard plains of the wrist were performed in all patients (anteroposterior, lateral, and oblique).

### Image interpretation

#### Radiography

All radiographs were evaluated by a board-certified radiologist and the referring hand surgeon. Localization of radiologically detectable pathologies was classified into five anatomical regions: distal radiocarpal/radioulnar, midcarpal, carpometacarpal, metacarpophalangeal, and proximal interphalangeal (PIP) and distal interphalangeal DIP joints. The reader concentrated on describing and counting the relevant lesions, e.g., non-relevant lesions like subtlely rippled articular surfaces were not reported.

#### Planar bone scan and SPECT/CT

Planar scintigraphy as well as SPECT/CT was each read by a different dual board-certified nuclear medicine physician and radiologist. All imaging (including standard radiographs) was read independently by the three different readers without knowledge of the results of the other imaging. In cases of disagreement concerning lesion evaluation and localization, the readers agreed on the lesions to be evaluated in consensus. Early-phase as well as late-phase planar images of the wrist and the hand were graded qualitatively (detection of focal, pathological uptake). Furthermore, the abovementioned anatomical regions were assigned as well. Radioisotope uptake on the SPECT/CT images was again graded qualitatively, and focal pathological uptake was assigned to the anatomical regions mentioned above, too. On combined SPECT/CT, detection of the pathology was based on the detection of osseous pathologies on CT (e.g., sclerotic areas, residual fissures, post-traumatic bone deformities, erosions) in conjunction with the abovementioned increased uptake. In cases of findings on the flat-panel CT component without increased uptake in SPECT, the findings were evaluated based on CT criteria only (sclerosis, residual fissures, etc.; see above). In cases of elevated uptake on SPECT imaging without a pathological correlate on the flat-panel CT component, the pathology was considered positive and as probably too minor or too early to induce morphological alterations on CT. Thus, the conclusion of the SPECT/CT imaging analysis was derived from a combined (morphological and metabolical) approach. SPECT/CT findings were then compared with the plain radiographs, with planar bone scan findings as well as plain radiographs *plus* planar bone scan findings read combined. This approach was chosen to compare SPECT/CT with imaging procedures before SPECT/CT approached clinical routine. SPECT/CT findings were furthermore correlated with the clinical diagnosis derived from clinical evaluation and planar radiographs. The readers concentrated on describing and counting the lesions which were suspected to be the clinically relevant ones in all imaging modalities.

#### Therapeutic influence

The final conclusion concerning the etiology of the pathologies as described above was based on complete clinical examination, imaging prior to SPECT/CT and follow-up (including - if available - surgical results) serving as the gold standard. If the SPECT/CT was negative and the clinical workup was negative, then SPECT/CT was evaluated to be true negative. When lesions were detected on imaging and the gold standard confirmed the pathology, the diagnostic imaging was evaluated to be true positive. Therapeutic impact was evaluated retrospectively in consensus with the referring hand surgeon based on the patients' charts. Initial diagnosis and treatment plan (as indicated in the clinical charts of the patient) prior to SPECT/CT was compared with the results and treatment plan after SPECT/CT imaging. When a conservative treatment plan was changed to surgery (and vice versa) as well as planned treatment was refrained because no pathology could be detected, those conditions were considered as change in therapy.

Statistically significant differences between the imaging modalities investigated here were evaluated by McNemar's test. A *p* value of <0.05 was considered statistically significant.

## Results

### Lesion detection

#### Plain radiography

A total of 48 pathologies were detected on plain radiographs. Fifteen lesions were located in the distal radiocarpal/radioulnar compartment, 14 in the midcarpal, 11 in the carpometacarpal, 2 in the metacarpophalangeal, and 6 in the PIP and DIP joints. All of these lesions were due to degenerations, e.g., sclerosis of joint surfaces, joint narrowing, and osteophytes. In five of these cases, additional posttraumatic or postoperative bone defects (e.g., removement of a midcarpal bone, persisting fractures) were detected.

#### Planar scintigraphy

**Figure 1:**
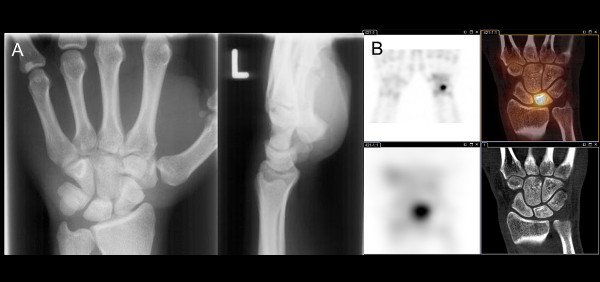
**A 25-year-old male with left-sided wrist pain when grabbing and lifting.** Tendinitis was suspected. (**A**) Plain radiographs showed no bone lesion. (**B**) SPECT/CT revealed an intense radioisotope uptake in the lunate bone corresponding with osteomalacia of the lunate bone followed by surgery (top row: three-dimensional (3D)-SPECT and fusion SPECT/CT, bottom row: planar SPECT and CT-alone).

**Figure 2:**
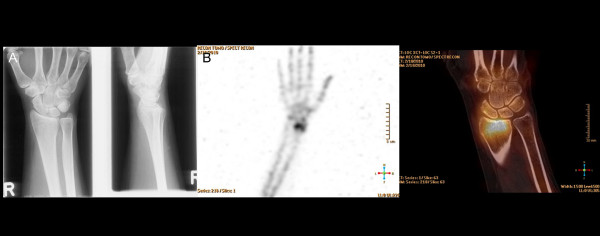
**A 32-year-old female with persistent dorsoradial wrist pain after distortion.** (**A**) No bone lesion was detected on plain radiographs. (**B**) High radioisotope uptake in the distal radial epiphysis with bumpy RC joint surface, representing persistent bone remodeling after consolidated distal radial fracture (left: 3D-SPECT, right: fusion SPECT/CT). Occupational therapy was initiated after SPECT/CT.

**Figure 3:**
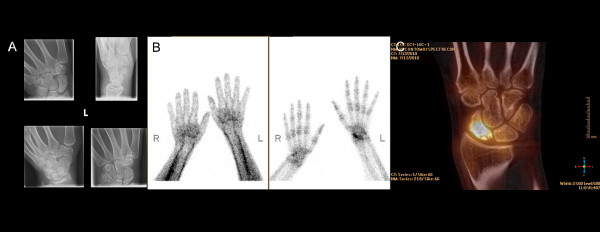
**A 28-year-old male with suspected RC osteoarthritis after scaphoid fracture 6 years ago.** The patient has a consolidated pseudoarthrosis of the scaphoid bone and persistent wrist pain. (**A**) Plain radiographs show focal sclerosis of the proximal pole of the scaphoid. (**B**) Planar bone scan revealed uptake in the scaphoid bone on late-phase images (left: early phase, right: late phase). (**C**) Coronal SPECT/CT images showed uptake in the proximal scaphoid (osseous remodeling) and a vital proximal scaphoid fragment. The patient received revisional osteosynthesis of the scaphoid.

On planar bone scan, a total of 117 pathologies were detected. Sixteen lesions were located in the distal radiocarpal/radioulnar, 38 in the midcarpal, 31 in the carpometacarpal, 5 in the metacarpophalangeal, and 27 in the PIP and DIP joints. In five patients, no pathological radioisotope uptake was found. Overall, planar scintigraphy detected significantly more lesions than plain radiography (*p* < 0.05; Figures 1, 2, and 3).

#### SPECT/CT

Overall, 142 lesions were found in SPECT/CT. On the concomitant high-resolution CT scan, 76 additional osseous lesions (e.g., cysts, joint space narrowing, ossicles) were detected. Compared to plain scintigraphy, in 76 lesions, the exact localization of radioisotope uptake was provided by SPECT/CT (Figure 3). Again, in five patients, no pathological radioisotope uptake was found. Overall, SPECT/CT detected significantly more lesions than plain radiography and planar scintigraphy (*p* < 0.05).

### Concordance of lesion detection

#### Concordance of lesion detection between X-ray vs. planar scintigraphy

In 15 patients, a concordance of lesion detection between X-ray and bone scan was found. No correlation concerning lesion detection could be established in the remaining 36 patients. Thus, planar bone scan revealed lesions with increased bone metabolism but without detectable lesions on plain radiography (Figures 1 and 2) in a significant number of patients.

#### Concordance of lesion detection between X-ray combined with planar scintigraphy vs. SPECT/CT

There was a concordance of lesion detection in 15 patients between X-ray and SPECT/CT and no agreement in 36 patients. However, when planar radiography *plus* planar scintigraphy were read combined, a concordance in imaging findings could be achieved in 27 patients. Thus, combined SPECT/CT revealed lesions and consecutive diagnosis in additional 24 patients.

### Correlation of clinical diagnosis and SPECT/CT findings

No correlation was found in 21 patients (40%, e.g., ulnar-sided pain but uptake and bony lesions radially or, e.g., midcarpal pain without pathological findings in this location). In 30 out of 51 patients, a correlation between the clinically suspected diagnosis and the SPECT/CT findings was found (60%), i.e., SPECT/CT confirmed the suspected diagnosis. Overall, skeletal pathologies (partly multiple lesions per patient) were classified as posttraumatic (12 patients), degenerative (20), inflammatory (3), and stress/overload (11) disorders. One patient had no skeletal pathology but had tendinosis of the flexor carpi ulnaris tendon (clinical and MRI diagnosis). In two patients, we found no pathologic disorder, but there was persisting bone growth. No radioisotope uptake was seen in five patients. Moderate/diffuse technetium uptake was seen in 12 cases, due to minimal bone matrix remodeling after trauma or based on minor osteoarthritic changes.

### Therapeutic influence of SPECT/CT

In 19 out of 51 patients (37%), SPECT/CT findings had significant impact on therapy. Treatment was changed or initiated after SPECT/CT diagnosis in 16 out of 51 cases (31%). Concerning the three remaining patients, therapeutic impact was given by excluding the clinically suspected diagnosis (Table 1).

**Table 1:** **Correlation of clinical and SPECT/CT findings with consecutive therapeutic change**

**Patients**	**Clinical symptoms**	**Estimated diagnosis**	**SPECT/CT diagnosis and therapy**
1	Persistent pain after arthroplasty CMC-1 and revision with partial resection of the trapezium bone	Possible persistent osseous reaction after arthroplasty	Significant uptake in MC-1 base and the trapezium bone Therapy: additional infiltration
4	Persistent posttraumatic wrist pain 6 months after undislocated radius fracture	Posttraumatic osseous lesion	Persistent posttraumatic reactive bone remodeling at fracture site, no significant osteoarthritis Therapy: additional occupational therapy
9	Unclear pain in CMC-1 with pain, swelling, and redness	Unclear, ganglion cyst (detected in MRI) not correlating with clinical symptoms	Mild uptake in the hamate bone and distal radius, no osteoarthritic changes Therapy: immobilization and additional occupational therapy
10	Unclear persistent wrist pain and pain in both Dig-I for several years	Osteoarthritis	No significant osteoarthritis, no signs of inflammatory arthritis Therapy: rheumatologic follow-up and supportive therapy
11	Unclear right-sided wrist pain (ulnocarpal)	Unclear, possible insertion tendinosis	No pathologic osseous findings, thus exclusion of possible diagnosis (clinically possible tendinosis) Therapy: immobilization, infiltration, and occupational therapy
16	Unclear pain in right CMC-1, no trauma	Unclear	Uptake in both STT joints, beginning (activated) osteoarthritis Therapy: occupational therapy
19	Arthritic right-sided wrist pain, no trauma	Triscaphoid osteoarthritis/possible irritation of the STT joint	Moderate bone remodeling of the right RC and SL joints without significant osteoarthritis, no STT osteoarthritis Therapy: occupational therapy, change of working place environment, and NSAID
20	Persisting left-sided wrist pain during flexion, no trauma	Unclear	Persisting bone growth, no bony lesion, exclusion of other differential diagnosis
22	Unclear right-sided radiocarpal wrist pain 1 year after trauma	Posttraumatic osseous/osteoarthritic lesion	Persistent bone remodeling at fracture site Therapy: occupational therapy
24	Radiocarpal right-sided pain for more than 1 year, no trauma	Unclear, MRI findings did not explain clinical symptoms	Elevated radiocarpal bone metabolism, together with clinical findings suggestive for tendovaginitis Therapy: immobilization, infiltration, and subsequent surgery
25	Carpal pain after scaphoid pseudoarthrosis, CT shows no persisting fracture	Triscaphoid osteoarthritis	Persistent bone remodeling in former fracture zone, no ST uptake but slight stress reaction radio-scaphoidal Therapy: surgery
29	Left-sided wrist pain while grabbing and lifting	Tendinitis	Osteomalacia of the lunate bone Therapy: surgery
34	Pain in the CMC-1 and MC-1 base, wrist trauma 20 years ago	Osteoarthritis STT joint	Posttraumatic intraosseous ganglion cyst in the right scaphoid Therapy: surgery recommended, yet not operated
36	Persistent pain after posttraumatic arthrodesis IP-I	CRPS	Persistent bone remodeling in arthrodesis, osteoarthritis CMC-2 joint, no CRPS Therapy: supportive and occupational therapy
37	Persitent pain in CMC-1 joints after trapezectomy and right arthroplasty >3 years ago	CMC-1 osteoarthritis	Severely activated CMC-1 osteoarthritis Therapy: revision surgery
46	Pain after scaphoid fracture and consolidated pseudoarthrosis	RC osteoarthritis	No RC osteoarthritis, vital scaphoid fragment with bone remodeling Therapy: revision surgery
49	Persistent pain in the distal ulna after distorsion >8 months ago	Ganglion cyst (diagnosed in MRI) was suspected to cause pain	Reactive elevation of bone metabolism at ulnar styloid (insertion of TFCC), ganglion cyst as the cause of clinical symptoms was excluded Therapy: immobilization, infiltration, and shaving during later course of disease
50	Carpal pain, known radiopalmar ganglion cyst	Possible additional STT osteoarthritis	Activated osteoarthritis in multiple IP and MCP joints, exclusion of STT osteoarthritis Therapy: ganglion resection
51	Left-sided carpal pain with painful bump	Carpal boss MCP III with reactive bony lesion and possible additional degenerative joint lesions	Carpal boss MCP-III with osseous reaction
			Therapy: surgical resection without additional arthrodesis (no additional joint degenerations)

CMC, carpometacarpal joint; CRPS, complex regional pain syndrome; IP, interphalangeal joint; MC, metacarpal joint; MCP, metacarpophalangeal joint; PIP, proximal interphalangeal joint; RC, radiocarpal joint; STT, scaphoid-trapezium-trapezoideum joint.

**Figure 4:**
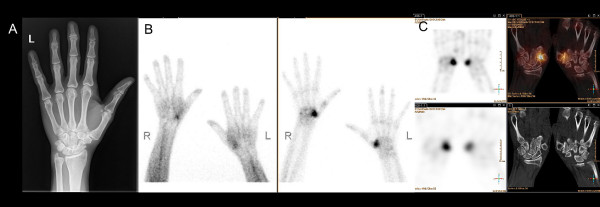
**A 48-year-old female with symptomatic bilateral CMC-1 osteoarthritis.** The patient had a history of trapezectomy on the left hand 3 years ago. (**A**) Plain radiographs showed moderate CMC-1 osteoarthritis on the left side. (**B**) Intense uptake in the right CMC-1 joint and moderate uptake in the left CMC-1 joint on early-phase imaging (left), intense radioisotope uptake in both CMC-1 joints on late-phase imaging (right). (**C**) SPECT/CT confirmed activated bilateral rhizarthritis/osteoarthritis with neoarticulation of the MC-1 and trapezoid bone on the right side. Patient received re-arthroplasty (top row: 3D-SPECT and fusion SPECT/CT, bottom row: planar SPECT and CT-alone).

Surgical treatment was subsequently performed in nine patients during the follow-up period (Figures 1, 3, and 4). Concerning the remaining patients, conservative treatment was initiated or amended. Surgery was recommended to at least one other patient, but the patient decided for conservative treatment. Overall, 38 out of 51 patients (75%) have been treated conservatively (Figure 2). In 32 patients, no therapeutic change was observed before and after SPECT/CT diagnosis. Concomitant uptake in 10 out of 51 patients (20%) who were asymptomatic in that specific location was detected on SPECT/CT. This was neglected clinically, thus false positive on SPECT/CT. These were mostly uptake in carpal joint spaces or uptake at the bony cortex/surface of carpal bones without any morphological correlate.

## Discussion

This is the first study to evaluate the diagnostic and therapeutic impact of SPECT/CT in patients with unspecific hand and wrist pain. This clinical situation is particularly challenging for the clinician because of the unsatisfying therapeutic options which can be offered to the patient. In this study, SPECT/CT proved not only to detect more lesions than clinical examination and conventional imaging prior to SPECT/CT, but also to detect metabolic and osseous changes which have been evaluated as the possible reason for the clinical symptoms. Thus, SPECT/CT might be integrated in a diagnostic algorithm as a problem solving tool when clinical examination and conventional imaging (plain radiographs, planar bone scan, as well as partly CT and MRI) fail to detect the cause of the patient's symptoms.

Currently, there are only a few studies available which investigated the diagnostic usefulness of SPECT/CT, partly compared with other imaging modalities. However, no other study has investigated this anatomical area in such a specific patient population, and none of the other studies investigated the specific therapeutic impact.

In a study by Hirschmann and co-workers, the value of SPECT/CT in patients with pain after surgical treatment of knee osteoarthritis was evaluated [6]. They found that SPECT/CT showed its highest value in establishing the suspected diagnosis for the clinician by displaying the specific anatomical location of the suspected pathology. Only a small number of patients were investigated. However, this basic ability of SPECT/CT, also in other specific anatomical areas, e.g., the hindfoot, has been investigated in several other studies as well [4,6,7]. Even-Sapir and co-workers found in a large cohort with 76 patients that SPECT/CT is able to detect significantly more lesions than conventional imaging workup [8]. Additionally, the specific localization of the metabolic activity was a major advantage concerning diagnostic confidence, thus potentially sparing additional imaging and costs. Improved diagnostic value was found to be as high as 58% concerning the final diagnosis. In comparison, in our study, a slightly smaller but more specific patient population was investigated, while the latter mentioned study investigated patients with nonspecific planar bone scan findings. Based on our highly selective patient population, the diagnostic improvement was not as high as in the latter study; however, the diagnostic improvement was in the range of other studies with SPECT/CT. Linke et al. conducted a large study with 71 patients with skeletal disorders and found that the addition of the CT information was followed by a diagnostic change in approximately one third of all patients in various skeletal conditions, e.g., re-classifications from osteoarthritis to fracture and suspected osteomyelitis to osteoarthritis [9].

In our study, SPECT/CT also detected significantly more lesions than plain radiography as well as planar bone scan, and the increase in lesion detectability yielded approximately 20% compared to planar bone scan and even more compared to plain radiography. In fact, CT revealed various bone disorders, such as joint narrowing, subchondral cysts, etc. However, often, osteoarthritic and postoperative changes do not cause pain. The metabolic aspect of SPECT/CT is able to demonstrate the focus of activated osteoarthritis or pathologic bone alteration. Additionally, we could confirm the abovementioned results that SPECT/CT offers more precise localization, and thereby, the clinically suspected diagnosis was confirmed in the additional 14% of our patients. However, we did not account this for therapeutic impact because the intended therapy has not been changed based on the SPECT/CT results. In another study by Hirschmann et al., the level of activation on SPECT/CT correlated significantly with the stage of osteoarthritis seen on plain radiographs [6]. However, no correlation was observed between the degree of bony deformation and the detected activity as well as between activation and signs of osteoarthritis on CT scans. Overall, even if there was (partly) severe bony deformation in keeping with morphological signs of osteoarthritis, the degree of morphological deformation did not correlate well with the clinical symptoms of the patients. Thus, the amount of uptake/level of activation was found to be more useful in the determination of the actual disease status than morphological imaging alone. In our study, radiographs were negative in several cases, while SPECT/CT found pathologies responsible for the pain syndrome. However, we additionally found several lesions where plain radiography or CT showed bony pathologies without increased uptake and/or there were additional, more subtle lesions which were found responsible for the clinical symptoms. This might be especially important for patients with severe osteoarthritic wrists (SLAC or SNAC) where it can be impossible to determine which of the numerous lesions is currently active by means of morphological imaging. SPECT/CT also has shown to be valuable in related indications, e.g., evaluation of bone vitality [3].

There are only very few studies currently available in the literature which evaluated the change in therapy based on SPECT/CT findings. In our study, we added the evaluation of therapeutic impact based on an interdisciplinary discussion between our institute and the department of hand surgery. As mentioned, in 19 out of 51 patients, significant influence on therapy was observed. Of these, SPECT/CT findings led to surgical treatment in the majority of patients. Especially in those cases, the prior imaging findings have been discreet on plain radiography. On conventional bone scan, the exact anatomical localization was difficult to identify, thus lacking a clear-cut diagnosis. Our results do not completely endorse the findings of the study of Gnanasegaran et al. where SPECT/CT provided additional diagnostic information in 81% of patients with unexplained foot pain or postoperative disorders [7]. Thereupon, patient management was changed in 62%. Those differences might be explained by the more standardized imaging procedures in our patient population as well as by the much more specific patient population. However, post-therapeutic evaluations might be an additional example where SPECT/CT might have a future in as a standard diagnostic tool, because MRI is often impaired in those cases due to prominent contrast media uptake post-operatively. According to the overview of Scharf, old and new injuries in these special cases only could be ruled out with SPECT/CT because the ability to localize activity within a bone or at an articular surface allows for differentiation between fractures and joint disease [10].

Our study has several limitations. Firstly, evaluating the therapeutic impact was conducted retrospectively. However, it was jointly evaluated together with the hand surgeons in our hospital, and it was evaluable based on the patients' charts which therapy was indeed changed based on the results of the SPECT/CT. SPECT/CT also had false positive findings in ten patients. These were neglected based on the clinical findings by the treating surgeon after discussion with the imaging specialist. Thus, SPECT/CT always has to be considered in a clinical context as a whole, and the clinical examination should certainly not be neglected when evaluating multimodality images. Not all patients had totally the same imaging scheme prior to SPECT/CT. All patients had X-rays and planar bone scan to compare with (from the combined SPECT/CT procedure), but only half of our study population had MRI. However, all patients had unspecific hand and wrist pain at the time point of the SPECT/CT. The results of the direct comparison of SPECT/CT vs. MRI concerning lesion detection and characterization have already been published [11]. In that study, we mainly found a higher sensitivity of the MRI concerning lesion detection, but a higher specificity of SPECT/CT in depicting the clinically relevant lesions. Thus, our study presented here concentrated on the diagnostic impact on therapy rather than on diagnostic accuracy. SPECT/CT, as a combined metabolical/anatomical imaging modality, is methodically impaired concerning soft tissue evaluation, e.g., tendons, sheaths, as well as fibrous tissue. However, new concepts already demonstrated that even in SPECT/CT, truly combined radiological/metabolical imaging is possible - even with intra-articular contrast media [12]. Radiation is certainly a topic not to neglect. However, in our study, we used a middle-dose CT with 80 mA in a FOV of 40 cm which can be considered a ‘justifiable’ dose, especially in the extremities.

## Conclusions

SPECT/CT proved to be superior compared to X-ray and planar bone scan concerning lesion detection. Additionally, integration of SPECT/CT into the diagnostic workup of this specific patient population showed influence on consecutive therapy in a significant number of patients. As a result of this study, SPECT/CT is now increasingly used in daily clinical practice for hand and wrist disorders in our institution. Our results encourage for further investigations with SPECT/CT to line out the higher lesion localization capabilities and its possible impact on therapy in other non-oncologic skeletal disorders. In a following study, SPECT/CT will be compared with MRI to investigate the (dis)advantages of each examination method in this patient population.

## Abbreviations

CMC: Carpometacarpal joint; CRPS: Complex regional pain syndrome; PIP: Proximal interphalangeal joint; RC: Radio-carpal joint; STT: Scaphoid-trapezium-trapezoideum joint.

## Competing interests

Klaus Strobel received an ISS grant from Philips Healthcare, Best, Netherlands, for other studies in SPECT/CT. However, data of those are not included in the study presented here. Patrick Veit-Haibach is a current recipient of ISS grants from Bayer Healthcare, Berlin, Germany. Those studies do not include SPECT/CT research. All other authors declare that they have no competing interests.

## Authors’ contributions

FS carried out the manuscript drafting, data collection, and data analysis. MS carried out the data collection, therapy evaluation, and data analysis. MWH carried out the manuscript drafting, data collection, and statistical analysis. UH carried out the data collection, therapy evaluation, and data analysis. UvW carried out the data collection, therapy evaluation, and data analysis. KS conceived of the study and participated in its design and coordination. PVH conceived of the study, participated in its design and coordination, carried out the final manuscript drafting, and is the senior responsible study person. All authors read and approved the final manuscript.
